# Automated breast volume scanner based Radiomics for non-invasively prediction of lymphovascular invasion status in breast cancer

**DOI:** 10.1186/s12885-023-11336-w

**Published:** 2023-08-30

**Authors:** Yue Li, Xiaomin Wu, Yueqiong Yan, Ping Zhou

**Affiliations:** Department of Ultrasound, The Third Xiangya Hospital, Central South University, Changsha, 410013 Hunan China

**Keywords:** Breast cancer, Lymphovascular, Automated breast volume scanner, Radiomics model

## Abstract

**Purpose:**

Lymphovascular invasion (LVI) indicates resistance to preoperative adjuvant chemotherapy and a poor prognosis and can only be diagnosed by postoperative pathological examinations in breast cancer. Thus, a technique for preoperative diagnosis of LVI is urgently needed. We aim to explore the ability of an automated breast volume scanner (ABVS)-based radiomics model to noninvasively predict the LVI status in breast cancer.

**Methods:**

We conducted a retrospective analysis of data from 335 patients diagnosed with T1-3 breast cancer between October 2019 and September 2022. The patients were divided into training cohort and validation cohort with a ratio of 7:3. For each patient, 5901 radiomics features were extracted from ABVS images. Feature selection was performed using LASSO method. We created machine learning models for different feature sets with support vector machine algorithm to predict LVI. And significant clinicopathologic factors were identified by univariate and multivariate logistic regression to combine with three radiomics signatures as to develop a fusion model.

**Results:**

The three SVM-based prediction models, demonstrated relatively high efficacy in identifying LVI of breast cancer, with AUCs of 79.00%, 80.00% and 79.40% and an accuracy of 71.00%, 80.00% and 75.00% in the validation cohort for AP, SP and CP plane image. The fusion model achieved the highest AUC of 87.90% and an accuracy of 85.00% in the validation cohort.

**Conclusions:**

The combination of radiomics features from ABVS images and an SVM prediction model showed promising performance for preoperative noninvasive prediction of LVI in breast cancer.

## Introduction

Breast cancer is the second leading cause of cancer-related deaths annually [1, 2] and ranked as the most prevalent malignancy in females. Its incidence and mortality rates have continued to rise, even in early-stage cases where mastectomy is performed, with a 30% distant metastasis rate within 10 years [3]. LVI refers to tumor cells present in the endothelial space surrounding an invasive breast tumor, and it plays a crucial role in breast cancer spread [4–6]. According to the American Joint Commission on Cancer (AJCC)/Union for International Cancer Control (UICC) guidelines (TNM classification, 7th edition), LVI serves as an independent prognostic factor for breast cancer, indicating a higher risk of lymph node metastases, neoadjuvant chemotherapy resistance, and unfavorable survival outcomes [7, 8]. Currently, postoperative histopathological methods are the gold standard for diagnosing LVI, and no effective preoperative noninvasive prediction methods exist.

Radiomics has emerged as a significant breakthrough in oncology [9]. This technique converts medical images into high-throughput features, providing clinicians with quantitative data on tumor biological characteristics that are not visible to the naked eye [10]. Although MRI-based radiomics has shown promising results in assessing the LVI degree of breast cancer (AUC value: 87.0%), more accurate models are still required for predicting LVI [11–13]. Currently, ultrasonography is the most accessible method for breast tumor screening. It plays a crucial role in detecting and diagnosing breast cancer, especially when assessing lymph nodes in various regions [14]. By combining ultrasound and radiomics techniques, S. Bove et al. [15] established a predictive model for the sentinel lymph node (SLN) status in breast cancer patients, providing a novel approach in the application of ultrasound and radiomics in breast imaging. An automated breast volume scanner (ABVS) offers a 3D reconstruction of the breast based on ultrasound images, providing more comprehensive information about breast masses [16–18]. ABVS-based radiomics prediction models have been successfully applied to differentiate malignant breast lesions from benign ones and to predict axillary lymph node metastasis [19]. However, the use of ABVS-based radiomics for non-invasive prediction of LVI status has not been reported, and its potential for predicting LVI status in early-stage breast cancer remains unclear.

In this study, we aim to develop a radiomics model using ABVS features to noninvasively predict LVI in breast cancer. The model can serve as a valuable adjunctive tool for clinical decision-making. Additionally, when combined with other predictive factors such as traditional ultrasound feature and receptor status, it holds the potential to effectively forecast LVI and assist in formulating clinical treatment strategies.

## Materials and methods

### Patients

**Fig. 1:**
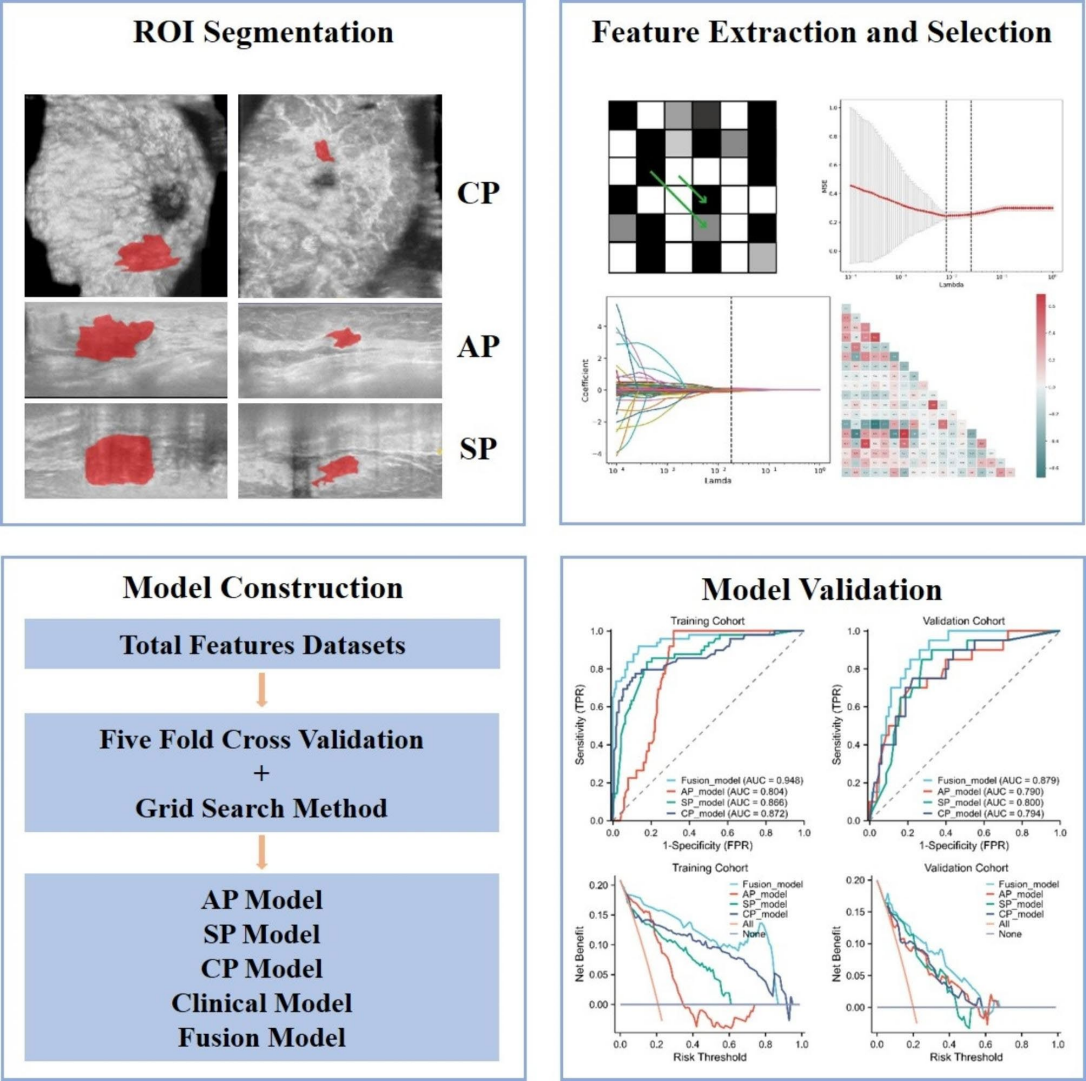
The workflow of this study, including tumor region segmentation, feature extraction, feature selection, model construction and evaluation

This study received approval from corresponding hospital’s Ethics Committee, and the need for written informed consent from patients was waived as it was a retrospective study. The workflow is depicted in Fig. 1. The study included 434 breast cancer patients who underwent ABVS examinations and subsequent surgery between October 2017 and September 2022. Inclusion criteria were: (I) confirmed breast cancer by pathology; (II) breast surgery completed within 2 weeks after ABVS examination; and (III) complete baseline data. Exclusion criteria were: (I) poor-quality or missing ABVS images; (II) bilateral breast cancer; (III) prior treatments (resection biopsy, neoadjuvant radiotherapy, or chemotherapy); and (IV) missing clinical or pathological data. The study finally comprised 335 patients.

### ABVS examination

The ABVS examination was performed using an ACUSON S2000 Automated Breast Volume Scanner (Siemens Medical Solutions, Inc. Mountain View, CA, USA) with a 14L5BV probe (bandwidth, 5–14 MHz). Patients were positioned supine or laterally with their arms above their heads. To ensure high-quality images, the scanning box was placed on the breast and gently pressurized after applying a uniform layer of medical gel. The scanning depth was adjusted according to breast size and target lesion location. Subsequently, the obtained axial plane (AP) and sagittal plane (SP) images were uploaded to a specialized workstation for image reconstruction, generating a coronal plane (CP) and a three-dimensional image.

### Pathological analysis

LVI-positive status was determined by immunohistochemistry (IHC), showing cancer cells infiltrating an endothelial-lined space around the peri-tumoral breast adenoma in breast cancer pathology. Further, IHC pathology confirmed estrogen receptor (ER), progesterone receptor (PR), human epidermal growth factor receptor 2 (HER2), and Ki-67 index. ER and PR were considered positive with ≥ 1% stained tumor cell nuclei. HER2 status was positive with IHC 3 + and negative with IHC 0 or 1+. IHC 2 + indicated an uncertain status, requiring fluorescence in situ hybridization (FISH) for gene amplification detection, and HER2 expression was positive if the ratio was ≥ 2.0. Axillary lymph node (ALN) positivity was present with the discovery of large or small metastases in one or more ALNs. For Ki-67 expression, the threshold was set at 30%, with < 30% indicating low expression and ≥ 30% indicating high expression.

### Region of interest delineation and radiomics analysis

The workflow of this study was showed as Fig. 1. Two experienced sonographers, one being senior, defined the region of interest (ROI) from AP images, prioritizing the largest section of breast cancer. For images with diverging tumor margins, the senior sonographer made the final ROI decision. ROIs from SP and CP images, also showing the largest tumor sections, were subsequently drawn. A total of 5901 features (1967 per plane) were extracted from ABVS images using the PyRadiomics package in Python 3.7.0. To increase feature diversity, Laplace-Gaussian and Wavelet filters were applied. These features included 14 shape features, 378 intensity statistical features, 504 Gy-level co-occurrence matrix (GLCM) features, 294 Gy-level dependence matrix (GLDM) features, 336 Gy-level run-length matrix (GLRLM) features, 336 Gy-level size area matrix (GLSZM) features, and 105 neighborhood gray-level difference matrix (NGTDM) features. Features with intraclass correlation coefficient (ICC) > 75% were considered reproducible and stable, and were retained for feature selection and machine learning model construction.

### Feature selection

Prior to feature selection, radiomics data were standardized, and sample balancing was achieved through 1:1 SMOTE to reduce bias in the training dataset [20]. Three feature selection pipelines were employed: (1) Mann-Whitney U test to identify the features with a significantly statistical value (p < 0.05) between the LVI group and non-LVI group; (2) LASSO with ten-fold cross-validation to select the most significant features; (3) The Spearman correlation coefficient analysis assessed feature correlation, retaining only the feature with a better AUC when the correlation coefficient was ≥ 80.00% or ≤ -80.00%. then the retained features were used to develop machine learning models. We also performed Boruta-Shap algorithm, which calculated feature importance through 100 iterations, to rank the selected features and illustrate their average Shapley values.

### Model construction and evaluation

We used support vector machine to construct three independent radiomic models for LVI prediction using the selected feature for each plane (AP, SP, CP). Hyperparameters of the models were optimized using grid search and cross-validation. The models’ prediction ability was visually demonstrated through ROC curves, AUC, and decision curves. Diagnostic ability was evaluated using accuracy, specificity, sensitivity, NPV, and PPV.

### Statistical analysis

We utilized SPSS (version 22.0) to calculate and analyze the data. For numerical data, we employed the independent-samples t-test [21], Fisher’s exact test [22], and Mann-Whitney U test to compare group differences [21, 22]. The chi-square test was used to analyze categorical variables in the grouped populations. Spearman correlation analysis was conducted to examine the correlations between each radiomics feature pair. To compare the differences in AUC between the prediction models, we used the Delong test. Statistical significance was defined as p < 0.05.

## Results

### Baseline characteristics

A total of 335 females with breast cancer (mean age: 51.5 ± 10.42 years; range: 27 to 82 years) were included. Among them, 69 (18.9%) showed positive LVI status, while 266 (81.1%) showed negative LVI status. The LVI + and LVI- groups exhibited significant differences in histologic type, Ki-67 status, and PR status (all p < 0.05). There were no significant differences in other characteristics (p = 0.092–0.919). Baseline data are presented in Table 1. After training cohort (n = 235) and validation cohort (n = 100) division, there was no significant differences in clinicopathologic characteristics between these two cohorts. Table 1 presents the baseline data of all patients.

**Table 1:** The clinicopathologic characteristics of all patients

Characteristics	Non-LVI group	LVI group	P value
n	266	69	
age, mean ± sd	51.01 ± 10.71	50.70 ± 10.45	0.829
BI-RADS, n (%)			0.639
3	2 (0.6%)	0 (0%)	
4	181 (54%)	47 (14%)	
5	80 (23.9%)	20 (6%)	
6	3 (0.9%)	2 (0.6%)	
convergence sign, n (%)			0.097
no	212 (63.3%)	61 (18.2%)	
yes	54 (16.1%)	8 (2.4%)	
Strain elasticity, median (IQR)	4 (4, 5)	5 (4, 5)	0.092
Tumor length, n (%)			0.349
< 2 cm	93 (27.8%)	20 (6%)	
≥ 2 cm	173 (51.6%)	49 (14.6%)	
SLNB, n (%)			0.479
no	97 (29%)	22 (6.6%)	
yes	169 (50.4%)	47 (14%)	
ER status, n (%)			0.919
negative	71 (21.2%)	18 (5.4%)	
positive	195 (58.2%)	51 (15.2%)	
PR status, n (%)			0.409
negative	83 (24.8%)	18 (5.4%)	
positive	183 (54.6%)	51 (15.2%)	
HER2 status, n (%)			0.197
negative	169 (50.4%)	38 (11.3%)	
positive	97 (29%)	31 (9.3%)	
Ki67, n (%)			0.018
negative	139 (41.5%)	47 (14%)	
positive	127 (37.9%)	22 (6.6%)	

Deviation: LVI, Lymphovascular invasion; BI-RADS, Breast Imaging-Reporting and Data System; IQR, Interquartile Range; SLNB, Sentinel Lymph Node Biopsy; ER, estrogen receptor; PR, progesterone receptor; HER2, human epidermal growth factor receptor-2; Ki-67, cellular proliferation index

### Feature selection

**Fig. 2:**
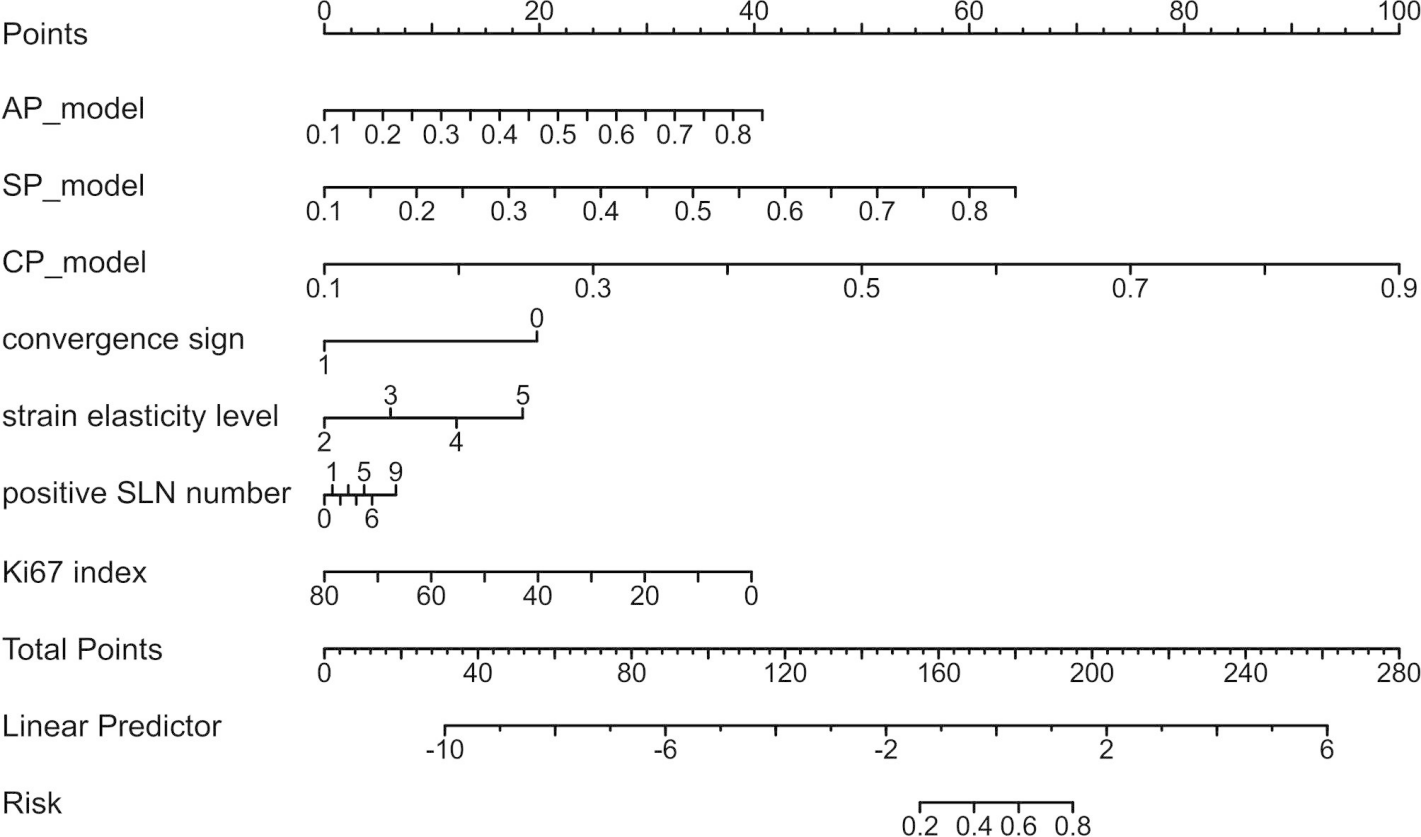
The nomogram integrating three single-modality radiomics signature and significant clinicopathologic factors to predict lynphovascular invasion

A total of 5658 radiomics features (ICC values > 75.00%) were extracted from AP, CP, and SP images of ABVS. After feature selection pipeline, 6, 5 and 6 radiomics features were retained for AP, SP and CP plane, respectively. Features with an absolute correlation coefficient value ≥ 80.00% were considered highly correlated, and the feature with a lower AUC was eliminated. The correlation coefficients between each feature pair are shown in the heat map. Those features were further used to develop distinct single-scale models for each plane. After univariate and multivariate logistic regression, 4 clinicopathologic characteristics (convergence sign, strain elasticity level, positive SLN number and Ki67 index) were selected for further analysis. The Fig. 2 showed the nomogram developed by multivariate logistic regression.

### Model construction and performance evaluation

**Fig. 3:**
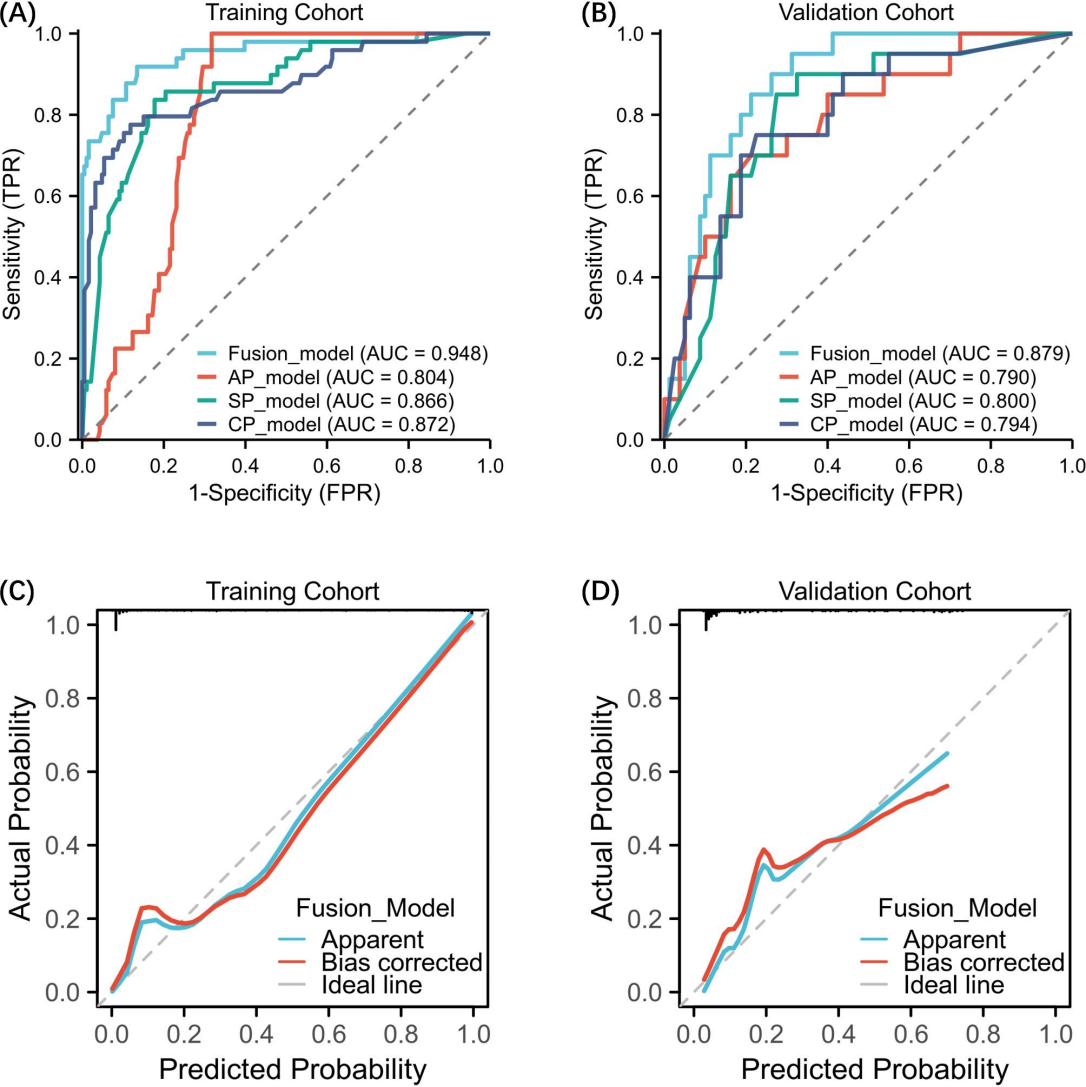
The ROC curves of different machine learning models in the training cohort (**A**) and validation cohort (**B**). And the calibration curves of the fusion model in the training cohort (**C**) and validation cohort (**D**)

**Fig. 4:**
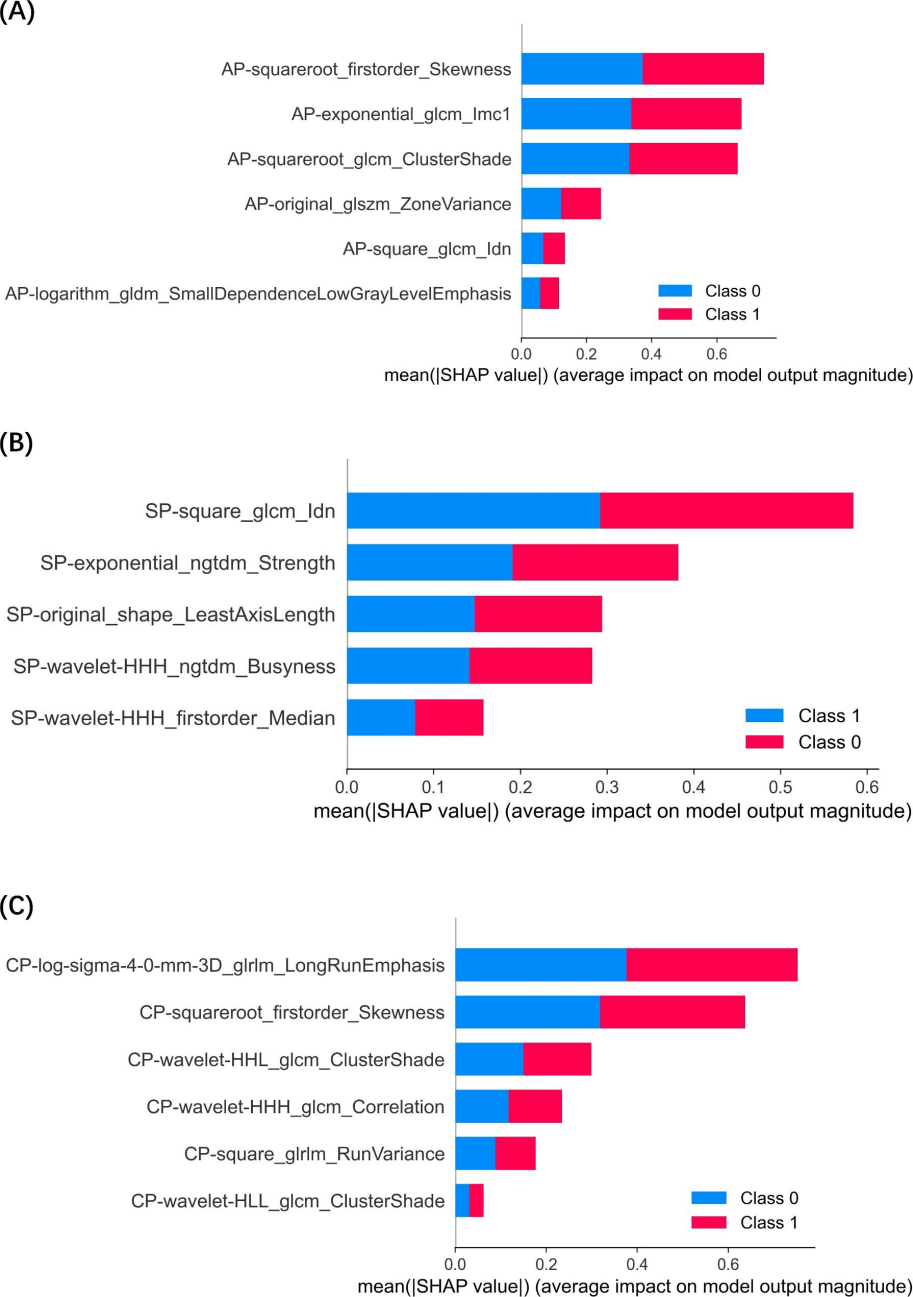
The feature importance ranking of the selected radiomics features assessed by a Boruta-Shap model for AP (**A**), SP (**B**) and CP (**C**) US image

**Fig. 5:**
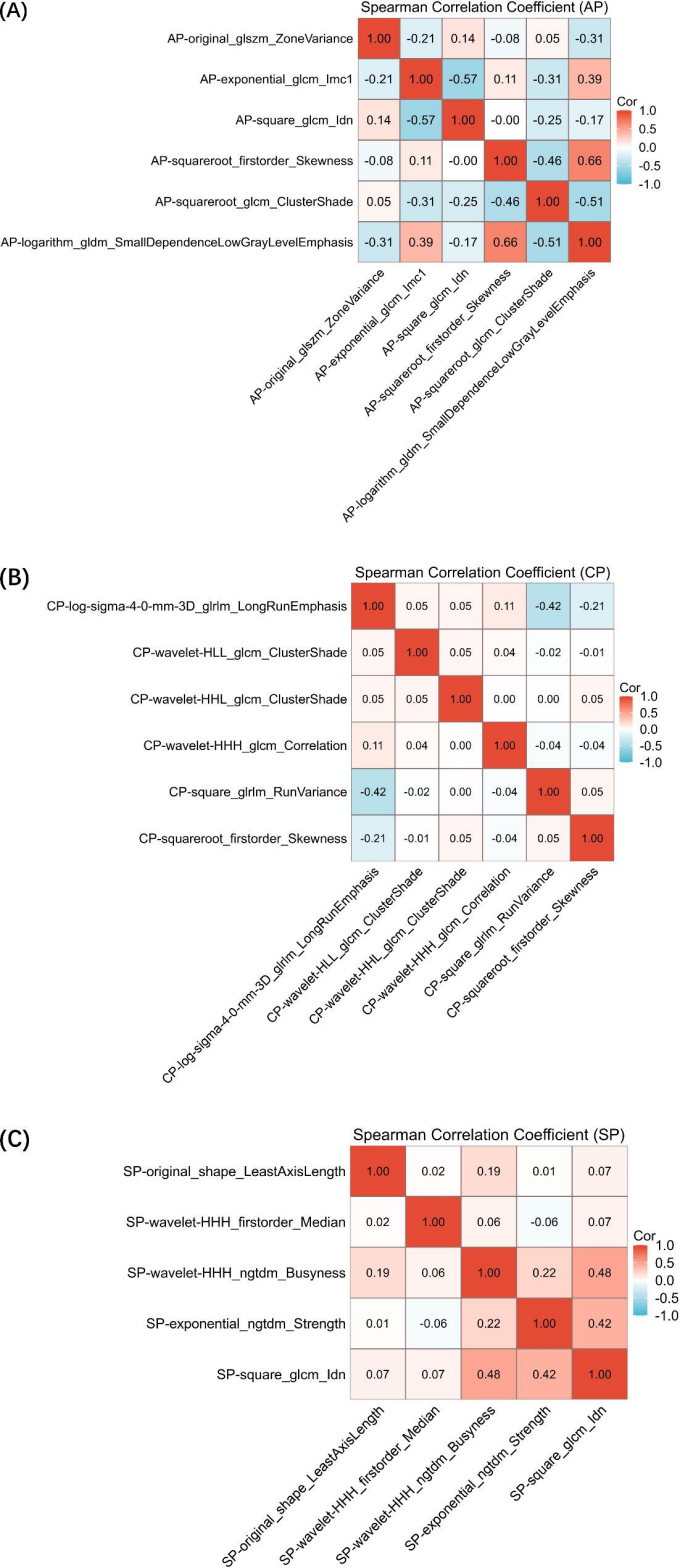
The Spearman correlation coefficient between any pair of radiomics features extracted from AP (A), SP (B) and CP (C) US image

We developed radiomics models to predict LVI status for each plane. The performance of each single-modality model (AP, SP, and CP) and their combination with clinicopathologic factors by stacking method are shown in Table 2. Fusion model achieved superior performance compared to single-scale models. The fusion model achieved an AUC of 94.80% (AP model: 80.40%; SP model: 86.60%; CP model: 87.20%) in the training cohort an AUC of 87.90% (pre-model: 79.00%; post-model: 80.00%; delta-model: 79.40%) in the validation cohort. DeLong test showed that fusion model significantly improved the model performance for predicting LVI status compared with the three single-modality models and the clinical model (all p < 0.05) in the training and validation cohort. The fusion model also performed well with an accuracy of 90.64% (AP model: 74.89%; SP model: 82.55%; CP model: 85.96%) in the training cohort, and an accuracy of 85.00% (AP model: 71.00%; SP model: 80.00%; CP model: 75.00%) in the validation cohort. The calibration curve of the fusion model also showed a great fit of the predicted results and ideal results. The Fig. 3 showed the different models’ performance and the calibration curve of the fusion model. We also used Boruta-Shap model to analyze the feature importance of the features, and the Fig. 4 illustrated the feature importance for each single-modality radiomics model. As the Fig. 5 showed, the radiomics features had no significant correlation between any pair of them, and it indicated that all the radiomics features were independent predictors for model.

**Table 2:** Performances of combining different machine learning models for predicting lymphovascular invasion in training cohort and validation cohort

Cohort	Model	AUC	ACC (%)	SEN (%)	SPE (%)	PPV (%)	NPV (%)
Training	Fusion model	0.95	90.64	83.67	92.47	74.55	95.56
	AP model	0.80	74.89	100.00	68.28	45.37	100.00
	SP model	0.87	82.55	83.67	82.26	55.41	95.03
	CP model	0.87	85.96	77.55	88.17	63.33	93.71
	Clinical model	0.65	68.35	59.18	70.74	34.52	86.93
Validation	Fusion model	0.88	85.00	70.00	88.75	60.87	92.21
	AP model	0.79	71.00	75.00	70.00	38.46	91.80
	SP model	0.80	80.00	65.00	83.75	50.00	90.54
	CP model	0.79	75.00	75.00	75.00	42.86	92.31
	Clinical model	0.58	70.00	50.00	25.93	86.96	86.96

The NPVs of single-modality model in predicting LVI status were 100.00% (AP), 95.03% (SP) and 93.71% (CP) in the training cohort, 91.80% (AP), 90.54% (SP) and 92.31% (CP) in the validation cohort. It indicated that the fusion model performed well in predicting ALN- cases. Considering that the model might performed differently for specific group, we also did sub-group analysis for molecular subtype, tumor length and BI-RADS level. For different molecular subtype, the fusion model achieved accuracies of 82.05%, 85.42% and 92.31% in HER2 + subtype, HR+/HER2- subtype and TN subtype, respectively, in the validation cohort. For different tumor length, the fusion model achieved an accuracy of 87.18% in < 2 cases and an accuracy of 83.61% in ≥ 2 cases, in the validation cohort. For different BI-RADS level, the fusion model achieved an accuracy of 82.67% in BI-RADS 4 cases and an accuracy of 92.00% in BI-RADS 5 cases, in the validation cohort. It indicated that the fusion model had robust and relatively high performance in different groups.

## Discussion

In this study, we utilized ABVS-based radiomics features to develop models for determining the LVI status in breast cancer. We applied robust feature selection pipeline to screen the ABVS radiomics features for each plane and integrated the radiomics signatures and clinicopathologic factors into a fusion model. Among these models, the fusion model, which incorporated all the features, demonstrated superior diagnostic performance in the validation cohort, with an AUC of 94.80% and an accuracy of 90.64%. Therefore, our ABVS-based radiomics model holds promise for noninvasively predicting the LVI status in early-stage breast cancer.

LVI is associated with an unfavorable prognosis in breast cancer patients and poses a high risk for ALN metastasis [4, 23]. Although in our study, more cases with ALN metastasis were found in the LVI + group compared to the LVI- group, we did not observe a significant difference between the two groups. This lack of significance may be attributed to the small positive sample size or the limitations of our current method for identifying LVI using H&E staining, which has reported inspection rates ranging from 9 to 50% [24]. Previous research has identified high Ki-67 expression (≥ 30%) and absence of PR as independent risk factors for LVI [25]; In our study, we also found a significantly higher proportion of high Ki-67 expression in the LVI + group compared to the LVI- group (p = 0.018). The presence of LVI and high Ki-67 expression collectively indicate an increased risk of breast cancer recurrence and metastasis.

Radiomics model had a higher performance than clinical model for predicting LVI in breast cancer. Different types of image plane may also impact the radiomics model performance. Table 2 shows that the clinical model had a lower AUC value (57.80%) than the any radiomics model (AP model: 79.00%; SP: 80.00% and CP: 79.40%). ABVS-based radiomics models had the similar accuracies in predicting LVI (AP model: 71.00%; SP: 80.00% and CP: 75.00%). Similarly, the fusion model’s AUC and accuracy based on the total features was higher than the single-modality model or clinical model. It indicated that any one of the planes may contributed to the machine learning model. To construct robust radiomics models, employing multi-modality imaging feature and might be vital. In this study, the total features screened by the same pipeline showed similar and robust prediction performance. The radiomics features from the three planes were complementary, indicating the best predictive power of the total feature. These findings highlight the trend in ABVS-based radiomics towards using multiple features to enhance model performance.

Our study had several limitations. Firstly, when examining the presence of LVI using H&E staining, we did not utilize endothelial markers like CD-34 and D2-40, which could potentially aid in detecting more LVI cases [24]. Secondly, to address the data volume difference between the two groups and reduce bias, we employed SMOTE, but this balancing process might have influenced the development of machine learning models. Thirdly, as a retrospective, single-center study, inherent biases and differences were inevitable. Additionally, our analysis solely focused on the radiomics features of ABVS, without incorporating pathomics and clinical omics data to establish a comprehensive model. For future studies, we plan to design prospective investigations, incorporate multi-omics data, and validate our model using external cohorts to enhance prediction accuracy.

## Conclusion

The ABVS-based radiomics model can precisely diagnose the LVI status of breast cancer and facilitate patient-specific treatment planning before surgery.

## Data Availability

The datasets used and/or analysed during the current study are available from the corresponding author on reasonable request.

## Abbreviations

ABVS: Automated breast volume scanner
ALN: Axillary lymph node
AP: Axial plane
AUC: Area under the curve
CP: Coronal plane
ER: Estrogen receptor
FISH: Fluorescence in situ hybridization
GLCM: Gray-level co-occurrence matrix
GLDM: Gray-level dependence matrix
GLRLM: Gray-level run-length matrix
GLSZM: Gray-level size area matrix
GNB: Gaussian naïve Bayes
H&E: Hematoxylin and eosin
HER2: Human epidermal growth factor receptor 2
ICC: Intraclass correlation coefficient
IHC: Immunohistochemistry
LASSO: Least absolute shrinkage selection operator
LR: Linear regression
LVI: Lymphovascular invasion
NGTDM: Neighborhood gray-level difference matrix
NPV: Negative predictive value
PPV: Positive predictive value
PR: Progesterone receptor
RF: Random forest
RFE: Recursive feature elimination
ROC: Receiver operating characteristic
ROI: Region of interest
SP: Sagittal plane
SVM: Support vector machine
